# Computer Tomography-Guided Percutaneous Indocyanine Green Injection for Intraoperative Mapping of Metastatic Suspected Lesions

**DOI:** 10.3389/fmed.2018.00191

**Published:** 2018-07-10

**Authors:** Anne Kreklau, Ruben Lopez Benitez, Jürgen Fornaro, Gesine Meili, Andreas Günthert

**Affiliations:** ^1^Department of Obstetrics and Gynecology, Cantonal Hospital of Lucerne, Lucerne, Switzerland; ^2^Department of Radiology, Cantonal Hospital of Lucerne, Lucerne, Switzerland

**Keywords:** Indocyanine Green, ICG, mapping metastatic suspected lesions, gynecological metastatic suspected lesions, gynecological oncology

## Abstract

**Introduction:** Surgical treatment in oncology is one of the main part concerning the surveillance rate of the patient in case of tumor recurrence. Metastatic suspected lesions are mostly located in the abdomen or pelvis and are diagnosed by PET, MRI, or CT scan. Especially surgery of small lesions in recurrent disease for diagnostic or therapeutic purpose is often challenging.

**Material and Methods:** We report a case series of 3 patients who were treated in our department due to a metastatic suspected lesion in PET-CT in follow up. For histological confirmation we performed a laparoscopy using a near infrared camera (NIR) for an improved visualization of the metastatic suspected lesion during surgical treatment. Previously the lesion was marked with an amount of Indocyanine Green (ICG) via computer tomography-guided percutaneous injection. The lesion was identified via NIR camera. While changing the camera in NIR mode, it show up as a blue spot due to the fluorescent signal. After correct identification it was removed and send to pathology.

**Results:** In all 3 cases they confirmed the diagnosis of a metastatic lesion. Complication occur in just one case, where the metastatic lymph node infiltrated the external iliac vein, which led to a high blood loss. In this case a vascular interposition had to be done.

**Conclusions:** Because of separate wavelengths, which are used for illumination and recording, only the marked area is visible, not the background.Due to correct identification, resection of the lesion was improved and healthy surrounding tissue could have been spared.

## Problem

**Figure 1:**
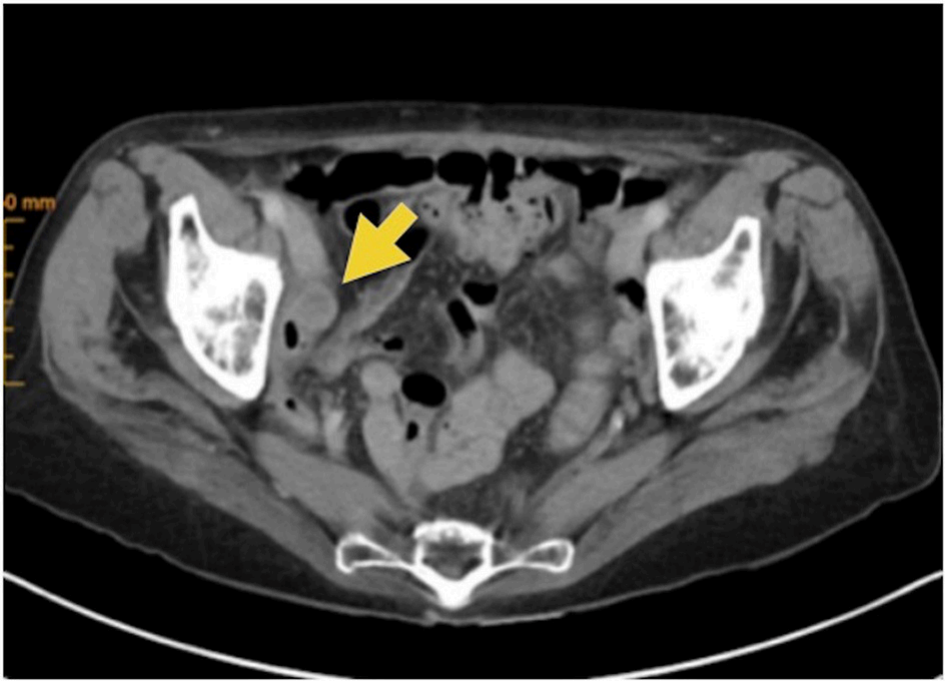
Metastatic suspected lesions in CT scan (marked with arrow).

Metastatic suspected lesions are mostly located in the abdomen or pelvis and are diagnosed by PET, MRI or CT scan (Figure 1). Especially surgery of small lesions in recurrent disease for diagnostic or therapeutic purpose is often a challenge, caused by 2 problems. First, detection of the suspected lesion is very difficult due to altered anatomy and adhesions caused by the operation or radiation. Second, intraoperative findings often do not correlate with imaging findings, in particular very small lesions. To detect a suspected lesion correctly while surgical treatment, an injection of a fluorescent dye, like ICG is possible. This attractive method to facilitate the visualization of lymphatic vessels, sentinel nodes, and metastatic lymph nodes has been introduced by Lim and Soter (1).

As a tracer Indocyanine Green is mostly used for SLN mapping in Cervical and Endometrial Cancer and showed increased detection rates compared to the Conventional Dyes (blue dye, 99Tc, or combination of the two tracers) (2, 3). Because of separate wavelengths, which are used for illumination and recording, only the marked area is visible, not the background. Extremely small concentrations are needed to make the marked area visible (4, 5).

## Our solution

In our department we treated three patients due to metastatic suspected lesion in the abdomen/pelvis caused by cervical cancer (2) and carcinosarcoma (1). The first patient was a 35-year old women with a metastatic suspected lesion 2 years after cervical cancer. Initial surgical treatment on FIGO IIA was performed as Total Mesometrial Resection with sentinel lymph node biopsy and was followed by radiation and chemotherapy. The lesion was located on peritoneal site paracolic left. It was resected without difficulties after mobilization of the colon descendens. In this case an expander was inserted to protect the colon in radiotherapy.

The second patient was a 30-year old women with metastatic suspected lesion near external iliac vein 2 years after cervical cancer. Initial surgical treatment on FIGO IB1 was performed as extended cervical conization with sentinel lymph node biopsy, pelvic lymphadenectomy right and paraaortic lymphadenectomy and was followed by radiation and chemotherapy. The lesion was marked on the left side without complication. Due to infiltration of the external iliac vein a severe bleeding was caused by trying to dissect the lesion. Therefore a laparotomy had been performed to stop bleeding. Patientin three, a 74-year old women, had a suspected lesion near external iliac vein 2 years after carcinoasarcoma. Initial operation was performed on FIGO IA by hysterectomy, adnexectomy, pelvic and paraaortic lymphadenectomy and omentectomy. No radiation or chemotherapy has been followed. In this case we performed a prophylactic clamping of the external iliac vein to avoid a mayor complication. The vein was blocked with bulldog-clamps. The operation was performed laparoscopically without any complications. This case is demonstrated in the video.

No ICG was used prior to mark the lymphnodes. Therefore we used ICG to mark the metastatic suspected lesion computer tomography guided in all three cases.

**Figure 2:**
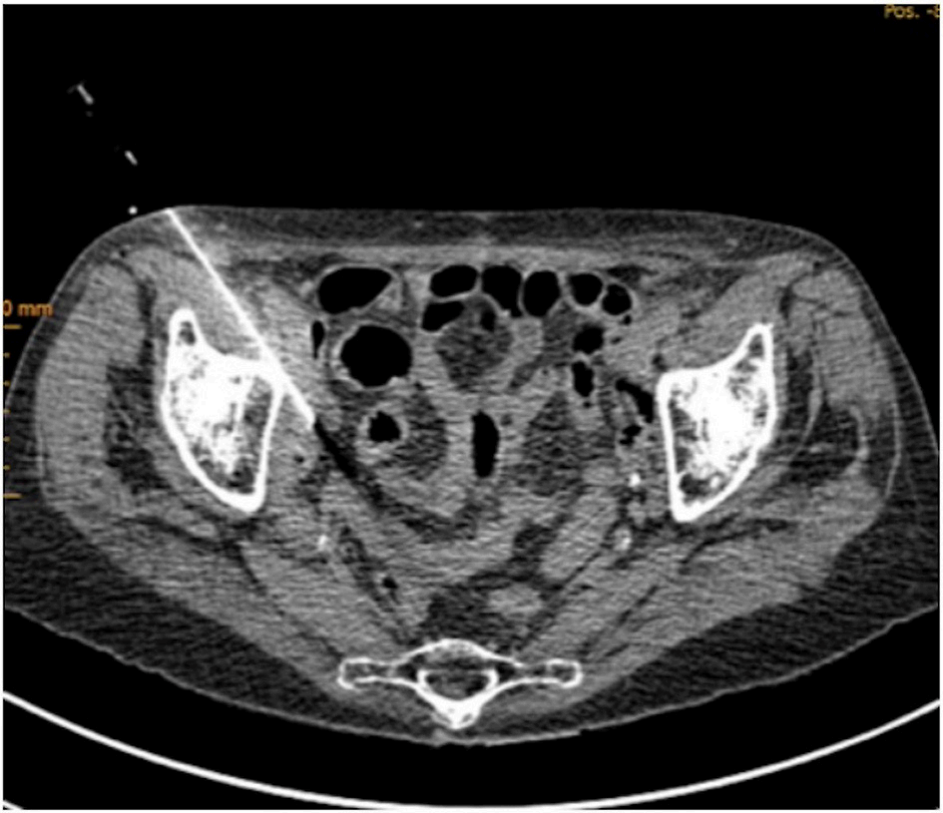
Computer tomography guided ICG injection into the suspected lesion.

One vial of 25 mg ICG powder was suspended with 5 ml of sterile water to get a maximum of concentration. We tried to insufflate as less as possible due to little capacity of the lymph node. After running a conventional CT with contrast agent first to demonstrate the lesion, 0.2–0.3 ml of ICG was injected into the lesion via 20-Gauge needle computer tomography guided (Figure 2). The patient was directly transferred to the operating room. The operation started 30 min after injection.

**Figure 3:**
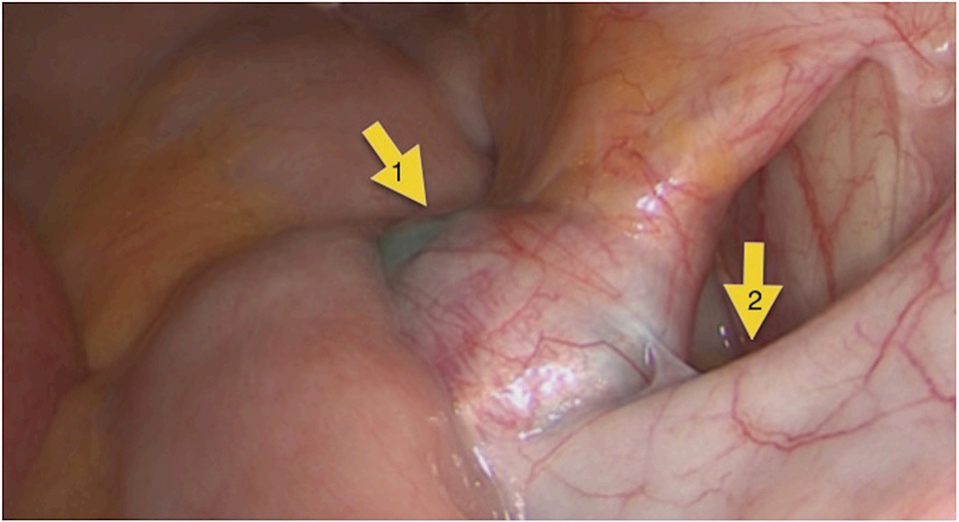
Situs intraoperatively: metastatic suspected lesion (1), external iliac vein (2).

**Figure 4:**
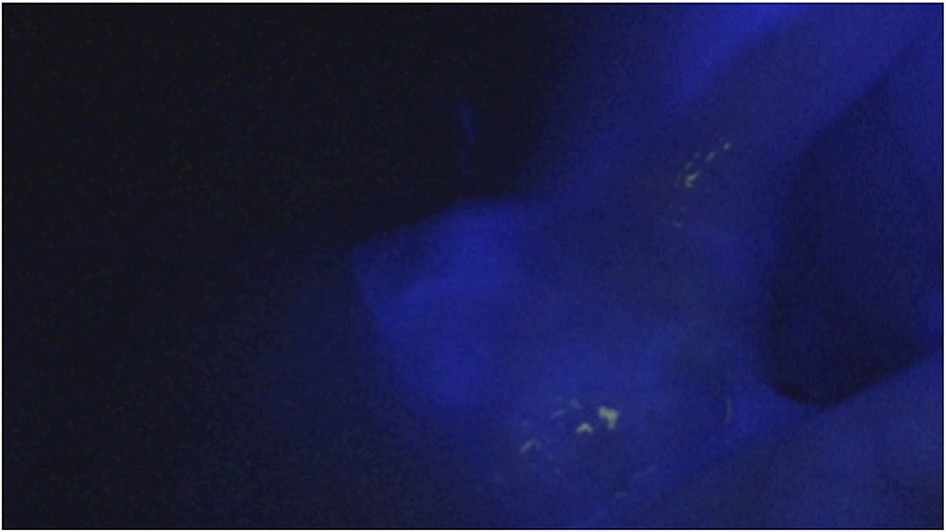
Illumination of the suspected lesion in infrared mode.

We performed a conventional laparoscopy with a NIR camera. In all three cases the suspected lesion show up as a blue spot (Figures 3, 4). The diagnosis of a metastatic lesion was confirmed by pathology in all three patients.

Complication occur in just one case, where the metastatic lymph node infiltrated the external iliac vein, which led to a high blood loss. In this case a vascular interposition had to be done.

## Discussion

In our video we are explaining how it is possible to detect small lesions laparoscopically after preoperative computer tomography guided Indocyanine Green injection. In a case series of 3 patients we were able to demonstrate an effective method of correct excision. Especially in the age of modern hybrid operating theaters, which are not yet in routine use in gynecology, a preoperative computer tomography guided injection with a fluorescent dye is a possible method which may lead to shorter operation times and less trauma by correct identification of the suspect lesion. Despite the fact that we do know the limitation of our case series, we truly believe that this cases could pave the way for the establishment of a new operation procedure.

## Ethics statement

All subjects involved in this case series gave written informed consent in accordance with the Decleration of Helsinki.

## Author contributions

AK and AG conception or design of the work and drafting the article. AK, GM, RL, and JF data collection. AK, GM, and AG data analysis and interpretation. AK, RL, JF, GM, and AG critical revision of the article. AK, RL, JF, GM, and AG final approval of the version to be published.

### Conflict of interest statement

The authors declare that the research was conducted in the absence of any commercial or financial relationships that could be construed as a potential conflict of interest.
